# The Virtual Electrode Recording Tool for EXtracellular Potentials (VERTEX) Version 2.0: Modelling in vitro electrical stimulation of brain tissue

**DOI:** 10.12688/wellcomeopenres.15058.1

**Published:** 2019-02-01

**Authors:** Christopher Thornton, Frances Hutchings, Marcus Kaiser

**Affiliations:** 1 Interdisciplinary Computing and Complex bioSystems (ICOS) Research Group, Newcastle University, UK, Newcastle upon Tyne, NE4 5TG, UK; 2 Institute of Neuroscience, Newcastle University, UK, Newcastle upon Tyne, NE2 4HH, UK

**Keywords:** Electrical Stimulation, Local Field Potential, Neocortex, Synaptic Plasticity, Spike-timing Dependent Plasticity, Short Term Plasticity, Long Term Potentiation

## Abstract

Neuronal circuits can be modelled in detail allowing us to predict the effects of stimulation on individual neurons. Electrical stimulation of neuronal circuits *in vitro* and *in vivo* excites a range of neurons within the tissue and measurements of neural activity, e.g the local field potential (LFP), are again an aggregate of a large pool of cells. The previous version of our Virtual Electrode Recording Tool for EXtracellular Potentials (VERTEX) allowed for the simulation of the LFP generated by a patch of brain tissue. Here, we extend VERTEX to simulate the effect of electrical stimulation through a focal electric field. We observe both direct changes in neural activity and changes in synaptic plasticity. Testing our software in a model of a rat neocortical slice, we determine the currents contributing to the LFP, the effects of paired pulse stimulation to induce short term plasticity (STP), and the effect of theta burst stimulation (TBS) to induce long term potentiation (LTP).

## Introduction

As an investigatory tool, electric field stimulation has facilitated many important experiments in neurophysiology and is still widely used. It is often used to provoke a population of neurons to fire simultaneously, producing a synaptic response measured in the local field potential (LFP). Alternatively, electrical stimulation can be used as a tool for neuromodulation using open- or closed-loop feedback to change the dynamics of neuronal circuits ^1^. However, interpreting this response potential is made difficult by the large number of possible synaptic sources, as well as by the diverse way in which the field stimulation recruits neurons of various morphologies. This extension to the VERTEX simulator ^2^ aims to combine our current knowledge of the neocortical microcircuit (neuron morphologies, patterns of connectivity, and synaptic properties) with a biophysical model of extracellular electrical stimulation and LFP generation to make a well informed prediction of the synaptic sources contributing to the electrically evoked LFP. As field stimulation experiments often seek to measure or manipulate synaptic efficacy, we include both short term plasticity (STP) and spike time dependent plasticity (STDP) in this release. We begin by describing how we incorporate the effect of an electric field on the membrane potential of the neuron compartments in our model. This follows the work of Frank Rattay ^3– 6^ and the implementation of the extracellular mechanism in the Neuron simulator ^7^. We then look at the two models of short term synaptic plasticity ^8, 9^ and spike timing dependent plasticity ^10^ previously described by others but included in this release, as well as details on their implementation. To illustrate how one may use our tool we look at the overall workflow involved in setting up a simulation including a stimulating electrode and synaptic plasticity and then describe an example simulation of stimulation in rat neocortex. We describe how we calculate the electric potential produced by a bipolar electrode equivalent to those typically used in *in vitro* experiments. This stimulation is then applied to the ongoing dynamics of a typical VERTEX simulation. In this case, we outline a model of rat neocortex based on the anatomy and physiology detailed at the Neocortical Collaborative Portal ^11^. We show how VERTEX ^12^ can be used to isolate the synaptic and non-synaptic changes that contribute to the change in response LFP during paired pulse stimulation and how theta burst stimulation causes STDP mediated changes in synaptic strength.

## Methods

### Simulating the effect of electric fields on neuronal fibers

Simulating electric field stimulation involves two steps: the first is to calculate the electric potential caused by the field we are interested in modelling, the second is to calculate how this affects the neuronal activity. The first step can be done analytically for simple electrode-tissue geometries, but for more complicated geometries must be solved numerically. We have provided an interface to models constructed using using the MATLAB Partial Differential Equation toolbox ^13^. These can be built to model a wide range of electrode-tissue setups, from bipolar penetrating electrodes used *in vitro* to non-invasive setups that include the skull and cerebrospinal fluid. VERTEX allows users to easily link to a model created using the PDE toolbox or to define an electrode setup analytically, by specifying an electrode location and equation to use. From these, an electric potential is calculated at the mid-point of each neuron compartment which is then used in the second step of the process. When modelling neurons using the multicompartmental approach first outlined by 14, the second step involves considering this extracellular electric potential when calculating the neuron membrane potential change of each compartment using the cable equation shown in Equation 1.

$$\frac{d(V_{i,n}-V_{e,n})}{dt}\cdot \;C_{m,n}+I_{ion,n}+\frac{V_{i,n}-V_{i,n-1}}{R_{n}/2+R_{n-1}/2}+\frac{V_{i,n}-V_{i,n+1}}{R_{n}/2+R_{n+1}/2}=0\;(1)$$

Where *V _i,n_* is the intracellular potential at compartment *n*, *V _e,n_* is the extracellular potential caused by the stimulating electrode at the mid-point of compartment *n*. *R _n_* is the resistance between compartment *n* and its neighbour, *C _m,n_* is the membrane capacitance at *n*, and *I _ion,n_* is the synaptic currents or other ion channel currents. The cable equation describes the flow of charge from one compartment to the other when their membrane potential differs. It is derived from Kirchhoff’s current law which states that current flowing into a particular node in a circuit must equal the current flowing out of that node. Normally when solving this we consider the extracellular potential to be constant across all compartments allowing us to ignore it. When it is not constant, it can be considered to contribute to the change in membrane potential. To do this we follow previous work ^6^ by introducing a reduced membrane potential *V* = *V _i_ − V _e_ − V _rest_* , to take into consideration the non-zero extracellular potential. Substituting this into Equation 1 and rearranging, we get Equation 2.

$$\frac{dV_{n}}{dt}=[I_{ion}+\frac{V_{n-1}-V_{n}}{R_{n-1}/2+R_{n}/2}+\frac{V_{n+1}-V_{n}}{R_{n+1}/2+R_{n}/2}+\frac{V_{e,n-1}-V_{e,n}}{R_{n-1}/2+R_{n}/2}+\frac{V_{e,n+1}-V_{e,n}}{R_{n+1}/2+R_{n}/2}]/C_{m,n}\;(2)$$

### Synaptic plasticity models

The efficacy of synaptic connections vary over time. Often these changes can be attributed to use-dependent plasticity—where the activity of the synapse and its constituent neurons determines the change. In VERTEX we take the synaptic efficacy to be the magnitude of the conductance or current depending on the synapse model type. So synaptic plasticity concerns the activity dependent changes in the conductance or current applied by a synapse.

**Short term plasticity (STP):** Short term plasticity has two components, facilitation (a short term increase in the efficacy of the synapse) and depression (a short term decrease). Both components are often present on the same synapse but the strength of one may mask the other ^15^. Short term depression occurs when the rate of replenishment of transmitter quanta is less than the rate of release; when a neuron endures sustained activation the replenishment of the transmitter-containing vesicles cannot keep up with their release, there is then less transmitter released and the postsynaptic response decreases ^16^. Short term facilitation has been attributed to an increase in the release probability caused by a build up of calcium ions in the presynaptic terminal ^17^, which then positively modulates local calcium channels ^18^. This release of VERTEX contains two commonly used short term plasticity models. One, which we refer to as the Abbott model, has been previously described and extensively used ^8, 19, 20^. It is a phenomenological model and unlike more detailed models of STP, it does not directly follow any biological mechanisms. However, it does reproduce key aspects of STP observed in the neocortex, can be parametrized by widely available measures, and can be implemented to run efficiently. The model contains two variables: *F* (the facilitation effect) and *D* (the depression effect) and four parameters: *f* the facilitation rate, *d* the depression rate, *tF* the facilitation decay rate, and *tD* the depression decay rate. *F* and *D* are both initially set to one, *f* should be greater than zero, and *d* should be between zero and one. When the presynaptic neuron generates an action potential each variable is updated according to the following rules:

F→F+f(3)

D→D⋅d(4)

The facilitation effect is increased by the facilitation rate ( Equation 3), and the depression effect is multiplied by the depression rate ( Equation 4). Like others ^8, 19^ we add rather than multiply the facilitation rate to avoid unrealistic facilitation during high frequency activity. At each time step, *F* and *D* are both subject to exponential decay ( Equation 5 and Equation 6).

$$F\to F+\frac{\left(1-F\right)}{tF}\cdot dt\;(5)$$

$$D\to D+\frac{\left(1-D\right)}{tD}\cdot dt\;(6)$$

The facilitation and depression effects multiply the synaptic weight as it is applied ( Equation 7, where *W _baseline_* is the weight defined for the synapse by the user). This allows synapse weights to depress to zero or to increase indefinitely under sustained firing and with the right conditions. *D* should always be one or less and decay back to one. *F* should always be one or more and decay back to one. The efficacy of the synapse at any given time is the original synaptic weight (a fixed conductance or current) multiplied by *F* and *D* ( Equation 7). *F* could be said to represent the level of calcium in the presynaptic terminal and *D* could represent the available quanta.

$$W_{applied}\to F\cdot D\cdot W_{baseline}\;(7)$$

The second model is known as the Markram and Tsodyks model ^9, 21^. It uses four variables. x, y, and z represent the fraction of resources available in recovered, active, and inactive states, with the resources available in the active state (y) determining the instantaneous strength of the synapses when a spike occurs. The fourth variable, u, represents the proportion of resources that will actually be used during an event (the proportion of resources moving from x to y). The variables update at each timestep according to Equation 8 to Equation 11. These are parametrised by *τ* (the time constant of the post synaptic current), *τ _rec_* (the time constant for recovery from synaptic depression), and *τ _fac_* (the time constant for facilitation).

$$x\to x+\frac{z}{\tau_{rec}}\cdot dt\;(8)$$

$$y\to y-\frac{y}{\tau_{I}}\cdot dt\;(9)$$

$$z\to z+(\frac{y}{\tau_{I}}-\frac{z}{\tau_{rec}})\cdot dt\;(10)$$

$$u\to u-\frac{u}{\tau_{fac}}\cdot dt\;(11)$$

As in the Abbott model there are also instantaneous updates that occur after a presynaptic spike. These are described in Equation 12 to Equation 14, with Equation 15 describing the synaptic weight applied. This weight will either be a conductance of a current depending on the model type.

u→u+U⋅(1−u)(12)

y→y+u⋅x(13)

x→x−u⋅x(14)

$$W_{applied}\to y\cdot W_{baseline}\;(15)$$

**Spike timing dependent plasticity (STDP):** The STDP implemented here is intended to model the NMDA receptor mediated plasticity that occurs on excitatory synapses. We use the classical pair-based exponential model previously described ^10^. According to this model, the change in efficacy of a synapse is a function of the relative arrival time of a presynaptic action potential at the synaptic terminal, and the generation of an action potential in the postsynaptic cell. When the presynaptic cell fires before the postsynaptic, the synapse strengthens, when the opposite occurs it weakens. Each synapse is specified by a pair of time constants, one for the postsynaptic neuron firing—that defines the decay of a potential weight decrease given a subsequent presynaptic spike— and one for the presynaptic neuron firing—that defines the decay of a potential weight increase given a subsequent postsynaptic spike. These potential weight changes can be seen as traces, and are stored as variables for each synapse, referred to as *A _pre_* and *A _post_* in the following equations. Equation 16 and Equation 17 are applied at each time step and show how *A _pre_* and *A _post_* decay according to their respective decay rates, *τ _Pre_* and *τ _Post_*.

$$A_{pre}\to A_{pre}-\frac{A_{pre}}{\tau_{pre}}\cdot dt\;(16)$$

$$A_{post}\to A_{post}-\frac{A_{post}}{\tau_{post}}\cdot dt\;(17)$$

As shown in Equation 18, the potential for negative weight change *A _post_* is increased instantaneously by a fixed amount ( *rate _post_* - a parameter specified by the user for each synapse model) when a postsynaptic spike occurs. The synaptic weight is also updated at this point by applying the addition of A _pre_, as shown in Equation 19.

$$A_{post}\to A_{post}+rate_{post}\;(18)$$

$$w\to w+A_{pre}\;(19)$$

Mirroring that for the post-synaptic spike, when a pre-synaptic spike occurs, the potential for positive weight change *A _post_* is increased instantaneously by a fixed amount *rate _pre_* when a postsynaptic spike occurs. The synaptic weight is then updated by the addition of *A _post_*, as shown in Equation 21. *A _pre_* is typically made positive by specifying a positive *rate _pre_* and *A _post_* made negative by specifying a negative *rate _post_*.

$$A_{pre}\to A_{pre}+rate_{pre}\;(20)$$

$$w\to w+A_{post}\;(21)$$

### Implementation

VERTEX is built using MATLAB, and makes use of the object-orientated and parallel programming support it provides. The core VERTEX program has been described previously ^2^, the changes made to VERTEX for this release comprise the addition of a mechanism to incorporate an extracellular stimulating field, and various forms of synaptic plasticity. This section will describe the various data structures used to store the properties and variables of the synapses and neurons, as well as the methods used to update the synaptic variables and weights during the main simulation loop.

**Figure 1.**
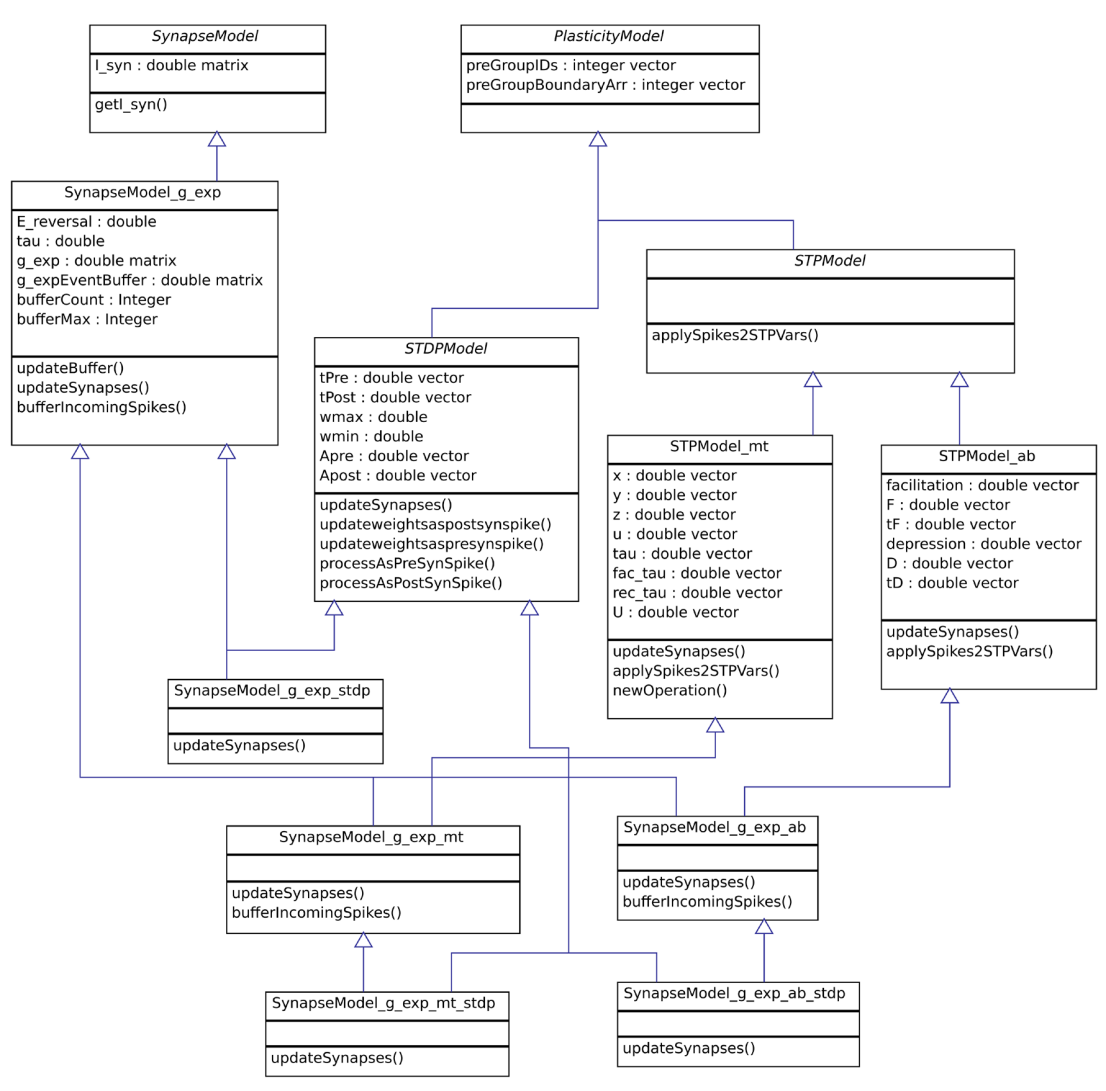
Using multiple inheritance to represent multiple types of synapse. The hierarchy of classes representing single exponential conductance based synapses, with and without spike timing dependant plasticity and short term plasticity. Other base synapse models (current based exponential, alpha, etc) are not shown here but fit in exactly as the SynapseModel_g_exp does.

**Class hierarchy:** Neuron and synapse types are described using inheritance to avoid the duplication of functionality. The abstract NeuronModel class describes the functionality provided by all multi-compartment neurons. It contains the membrane potential, external potential, and axial current (the currents that flow between compartments as a result of the difference between their membrane potentials) properties, as well as the functionality required to integrate these. The integration of Equation 2 is included as an additional step during the calculation of the axial currents and is performed at each time step when the stimulation is turned on. It is part of the core functionality of the abstract Neuron class. Classes with specific mechanisms then inherit from this, e.g. the NeuronModel_passive class provides a simple wrapper on top to allow a neuron with no active channels. The NeuronModel_adex adds the adaptive exponential integrate and fire mechanism to the soma, allowing the cell to generate action potentials. Here each instance of a class would represent a group of neurons in the same layer and of the same type. This allows us to ultilise MATLAB’s vectorised operations when updating variables so that for example: the membrane potential variable (v_m) holds the membrane potentials of all neurons in this group as a matrix. This also allows us to utilise the object oriented design advantages without the overhead that would come from storing each neuron or synapse as its own object. The integration of the axial current involves a loop over all possible neighbouring compartments with an operation vectorised for each compartment. The class hierarchy relevant to conductance based exponential synapses (SynapseModel_g_exp) is shown in Figure 1. Here, we have used multiple inheritance to allow us to efficiently define many combinations of synapse types. Synapse models have a base synapse type (defining how the synapse operates without plasticity, e.g. g_exp will be a conductance based exponential synapse), it can then also have short term plasticity (ab for the Abbott model or mt for the Markram and Tsodyks), spike timing dependent plasticity (stdp), or both. The plasticity models are defined as separate classes from which the synapse model can inherit from.

**Data structures for synapse variables:** Synapse models are required to provide a current to be applied to each neuron in the postsynaptic group. As such they store variables relevant to this calculation as vectors with an entry for each postsynaptic neuron. As STP model variables are dependent on the presynaptic firing we store these as vectors with an entry for each presynatpic neuron. This allows operations to be vectorised over all presynaptic neurons. In the STDP model *A _pre_* is a vector with an entry for each presynaptic neuron, and *A _post_* with an entry for each post synaptic cell, operations on these can be vectorised over all pre and post synaptic neurons respectively. Weight updates ( Equation 19 and Equation 21) can also be vectorised across either the postsynaptic or presynaptic neurons.

**Delays:** When modelling STDP it is important to consider delays - between the presynaptic neuron firing and the action potential reaching the synapse (axonal delay), and between the post synaptic neuron firing and the backpropagating action potential reaching the relevant part of the dendrite (dendritic delay) ^22^. As there is no vectorised solution to introduce dendritic delays, and as the axonal delay will dominate in most scenarios ^23^, we consider only the axonal delay. To incorporate this, we introduce a delay into the update rules for the weight change (Equations 19 and 21 become 22 and 23), Equation 20 and Equation 18 remain the same.

$$w(t_{post})\to w(t_{post})+A_{pre}(t_{post}-d)\;(22)$$

$$w(t_{pre}+d)\to w(t_{pre}+d)+A_{post}(t_{pre}+d)\;(23)$$

**Figure 2.**
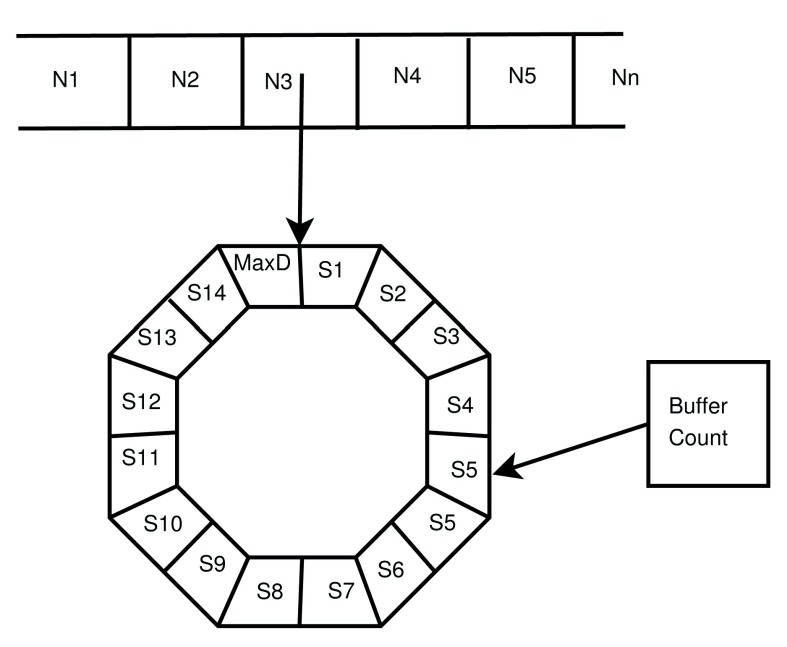
The data structure storing the *A _pre_* variable for the STDP model with delays. The first dimension of the array has an entry for each presynaptic neuron (N1 to Nn) the entry points to a unique circular array (buffer) with an entry for each delay step (S1 to the max delay steps, specified as a parameter). This stores the value of *A _pre_* for present and past time points. There is a buffer count that points to the location in the buffer that corresponds to the present.

Where *t _post_* is the time of the postsynaptic spike and *t _pre_* is the time of the presynaptic spike. To implement 22 we require access to past values of *A _pre_*, and so *A _pre_* becomes a two-dimensional array, so that each entry for each presynaptic neuron contains a circular array which stores a trace of *A _pre_* values. This is illustrated in Figure 2. Equation 23 cannot be vectorised over all postsynaptic neurons because the delay is inhomogeneous. Instead, we record a snapshot of activated synapses in a circular array. A buffer count points to the current location and the pre and post IDs of the activated synapse are placed into the array at *t _pre_* + *delay*. We can then vectorise the operation over all postsynaptic neurons that are receiving a spike at each time step. As including delays requires additional resources and is not always required we incorporate it in an additional STDP_delays class.

**Stimulation workflow:** To include electric field stimulation VERTEX, one must provide a model of the electric field. Essentially this must be able to describe the electric potential at the compartments of each neuron in the model. This can be achieved for fields which can be described analytically by passing the name of a function. This user defined function should be able to take a set of 3D coordinates and return a value for each of them describing the electric potential at that point. Another option is to use 3D modelling software such as Blender to build a 3D model of the tissue and electrode. This can then be imported into MATLAB as an STL (STereoLithography) file. The MATLAB PDE toolbox can then be used to calculate the electric field and potential across the tissue using geometry provided, and user input regarding the boundary conditions and volume conductor equation.

**Figure 3.**
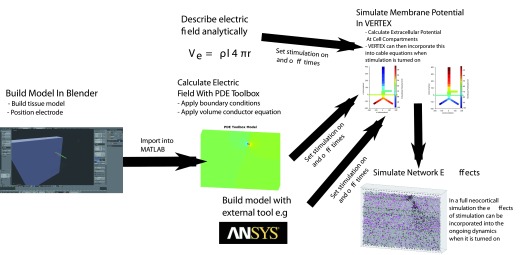
Workflow for including stimulating electrode in simulation. The user first specifies a stimulation field, this can be done by building a 3D model of the electrode and slice in blender, then using the MATLAB PDE toolbox to model an electric field across this. An analytic function describing a field is also an option, as well as models built using external tools such as ANSYS. The user then must also specify start and stop times for the stimulation, these are specified as a pair of lists containing times to start and stop, allowing multiple blocks of stimulation. VERTEX will then store the extracellular potential at the mid point of each neuronal compartment, and apply this during the simulation between the times specified.

**Figure 4.**
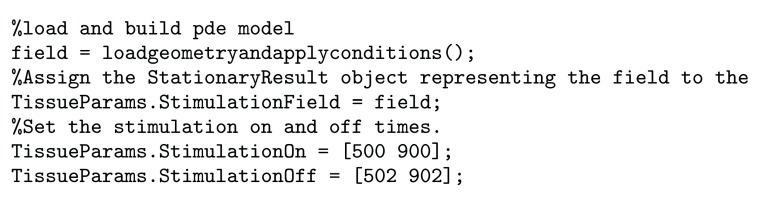
Code for assigning the stimulation on and off times. Applies two pulses each 2 ms long, one at 500 ms and one at 900 ms. This is part of a typical tutorial to run a simple network simulation in VERTEX.

The solution provided can then be passed to VERTEX as a StationarySolution object (part of the PDE toolbox) for static electric fields or a TimeDependentSolution object for time varying fields. However, other more powerful and flexible software solutions for constructing finite element models of electric fields exist, such as ANSYS ^24^ or COMSOL ^25^. Interface to these can be achieved by the user defined function described above, or by providing a grid of pre-calculated electric potentials at sufficient resolution so that MATLAB can interpolate from this to the midpoints of each neuron compartment. Ideally, users wishing to import from external tools should investigate the possibility of interfacing between their tool and MATLAB to allow their tool to calculate the values precisely at the compartment midpoints. Figure 3 shows the workflow involved in creating a VERTEX simulation with electric field stimulation. When the field has been calculated, VERTEX will calculate the electric potential at the midpoint of the compartment of each neuron. Users must also specify when the stimulation field is turned on and off, by specifying a StimulationOn and StimulationOff field in the TissueParams struct, as seen in Figure 4. For stationary fields, they will be applied for the duration of their on-time. Time-varying fields will be applied by looping through their time series at the same rate as the rest of the simulation, so that if they reach the end of their time course before they have been turned off they will continue again. This may save users time and memory as oscillating fields need only to be calculated for a one full cycle.

**Figure 5.**
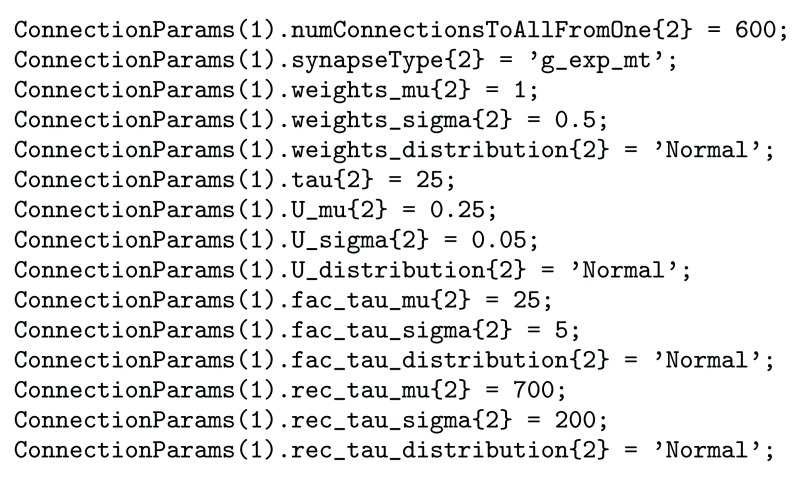
Shows the code required to add a synaptic connection from group 1 to group 2 with conductance based synapses with the Markram and Tsodyks model.

**Specifying synaptic parameters:** When building a simulation users will specify which synapse model to instantiate by providing the postfix of the class name (e.g. g_exp_mt). They will then also have to provide the parameters for the model. These can be specified either as a single value, or as a distribution. A single value will result in each synapse of this synapse group object having the same value, specifying a distribution will result in each synapse having a unique value drawn at random from the distribution. The distribution name should correspond to one of the names specified in the documentation of MATLAB’s makedist function (part of the Statistics and Machine Learning Toolbox). The user will also need to provide properties for the distribution such as mean and standard deviation for a normal distribution, which will also be found here. This allows a network to be compiled taking into consideration the natural variance found in these properties on a cell to cell basis. An example of how to set up and synaptic connection is shown in Figure 5, where we see how one specifies the distribution for each parameter, or in the case of *τ*, a single value.

**Requirements:** Stimulation using a model built with the PDE toolbox will require MATLAB 2016b or later, and the PDE toolbox. If users wish to execute code in parallel then MATLAB’s parallel computing toolbox is required. Hardware requirements depend on the size of simulation. Desktop computers can run simulations at the scale of a cortical column (<20K neurons), but a high performance computing node would be required for simulations of a full cortical slice (>100K neurons).

## Results

Here we illustrate the use of electric field stimulation in VERTEX with an example model of a bipolar stimulating electrode in a rat neocortical brain slice ^26^. We describe in detail the immediately evoked activity as well as the synaptic dynamics that result from repetitive stimulation, both in the short term (short term plasticity) and long term (spike-timing dependent plasticity). In doing so, we illustrate how one can create and incorporate a finite element model of the electric field created by a bipolar stimulating electrode into the VERTEX simulator to produce simulations of two experimental paradigms. We also seek to show the utility of the tool in revealing, based on anatomical and physiological data already obtained, the multiple currents contributing to the response, and how synaptic and neuronal dynamics may alter these under repeated stimulation.

### Generating the network

We build the network used in our simulation using knowledge of the local cortical microcircuit. Local circuit connectivity can be defined in terms of the cell-type and layer specific connection probabilities. These patterns influence the nature of spontaneous and evoked activity. Several studies have sought to reveal the local circuit connectivity by using anatomical or electrophysiological measures to create a map of connectivity probabilities between cell types and layers ^11, 27, 28^. These maps allow simulations of cortical dynamics to be embedded in an estimation of the anatomy of the cortical circuit. The implications that this measured anatomy has for the simulated dynamics can be seen in the activity within in each layer and each cell-type. For example, it has been shown that the experimentally measured anatomy of cat and rat neocortex ^27, 28^ implies the same flow of activity through cortex as that measured *in vivo* after transient thalamic stimulation ^29^. *In vitro* results have also been replicated, showing similarities in the properties of their model of gamma oscillations in macaque neocortex when compared with those measured in neocortical slices bathed in kainate to induce gamma oscillations ^2^.

**Figure 6.**
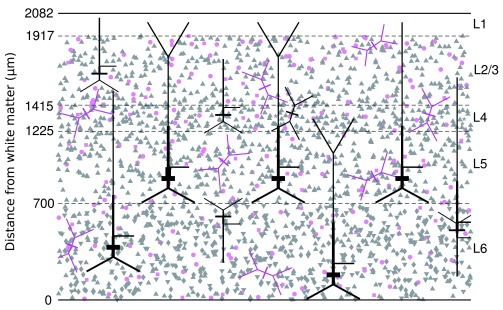
The structure of the neocortical slice model. The layer boundaries are shown as the dashed lines with the distance from the white matter given in *µ*m on the left. The rat somatosensory cortex has 6 layers, layer 1 contains no neurons and layers 2 and 3 have been combined. The position in the x and z planes of each neuron soma is shown, pink signifies inhibitory cells, grey signifies excitatory. The triangles are various types of pyramidal cell, stars are spiny stellate cells, circles are basket cells, and squares Martinotti cells. The full geometry of a selection of cell types are also shown.

**Figure 7.**
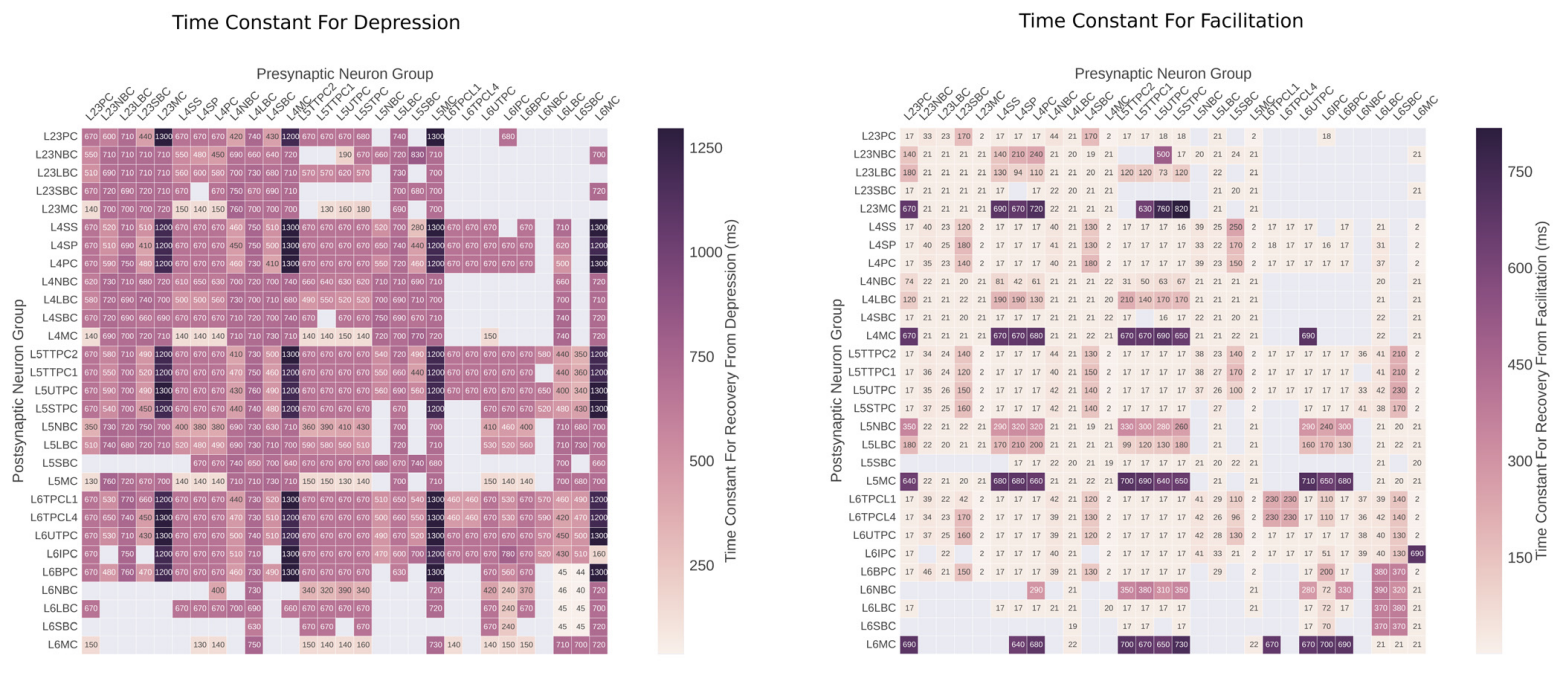
Time constants for facilitation and depression in rat neocortex, used for our model and taken from the Neocortical Microcircuit Collaborative Portal (NMCP) ^11^.

We construct our model of rat neocortex in VERTEX using the data from the the Neocortical Microcircuit Collaborative Portal (NMCP) ^11^. From here we take the neuron density, the neuron group types present and their proportions, the number of connections between the neuron groups, and their synaptic properties—synaptic strength and rates of facilitation or depression. The synaptic parameters for each neuron are randomly selected from a Gaussian distribution during the building of the model to account for the natural variation. In our model we refer to the plane adjacent to the white matter as the horizontal ( *X* dimension), the slice thickness as the depth ( *Y* dimension), and the plane from the white matter to the cortical surface as the vertical ( *Z* dimension). We model a typical *in vitro* neocortical slice preparation measuring 2000 *µ*m horizontally, 2082 *µ*m vertically x 400 *µ*m deep. It contains layers 1–6 but with layer one containing no neurons and layers 2 and 3 combined. The full slice model has a density of 103730 cells per mm ^3^ giving a total of 172773 neurons in the simulation. Figure 6 shows the layout of the slice, with sample geometries and the soma positions of 5% of neurons. Twenty-nine neuron types are included, defined by their layer of location, morphology, intrinsic dynamics, and connectivity. Table 1 shows the proportion of each neuron type within the model. Exponential, conductance based, synapses are used with the short term plasticity model from Markram and Tsodyks and spike timing dependent plasticity, the time constants used to parameterise these are shown in Figure 7.

**Table 1.** The 29 neuron types present in the model, along with their layer, their synaptic reversal potential (RP) indicating whether they are excitatory or inhibitory, and the proportion of the model they compose.

Neuron Group	Neuron Type	Layer	RP (mV)	Proportion
L23PC	Pyramidal Cell	2/3	0	0.1849
L23NBC	Nest Basket Cell	2/3	-70	0.0084
L23LBC	Large Basket Cell	2/3	-70	0.0143
L23SBC	Small Basket Cell	2/3	-70	0.0052
L23MC	Martinotti Cell	2/3	-70	0.0105
L4SS	Spiny Stellate	4	0	0.0128
L4SP	Star Pyramid	4	0	0.0345
L4PY	Pyramidal Cell	4	0	0.0841
L4NBC	Nest Basket Cell	4	-70	0.0030
L4LBC	Large Basket Cell	4	-70	0.0038
L4SBC	Small Basket Cell	4	-70	0.0019
L4MC	Martinotti Cell	4	-70	0.0037
L5TTPC1	Thick Tufted Pyramidal Cell	5	0	0.0630
L5TTPC2	Thick Tufted Pyramidal Cell	5	0	0.0765
L5UTPC	Untufted Pyramidal Cell	5	0	0.0108
L5STPC	Slender Tufted Pyramidal Cell	5	0	0.0630
L5NBC	Nest Basket Cell	5	-70	0.0063
L5LBC	Large Basket Cell	5	-70	0.0066
L5SBC	Small Basket Cell	5	-70	0.0007
L5MC	Martinotti Cell	5	-70	0.0124
L6TPC_L1	Tufted Pyramidal To Layer 1	6	0	0.0515
L6TPC_L4	Tufted Pyramidal To Layer 4	6	0	0.0453
L6UTPC	Untufted Pyramidal Cell	6	0	0.0546
L6IPC	Inverted Pyramidal Cell	6	0	0.1094
L6BPC	Bitufted Pyramidal Cell	6	0	0.0999
L6NBC	Nest Basket Cell	6	-70	0.0062
L6LBC	Large Basket Cell	6	-70	0.0146
L6SBC	Small Basket Cell	6	-70	0.0021
L6MC	Martinotti Cell	6	-70	0.0106

**Figure 8.**
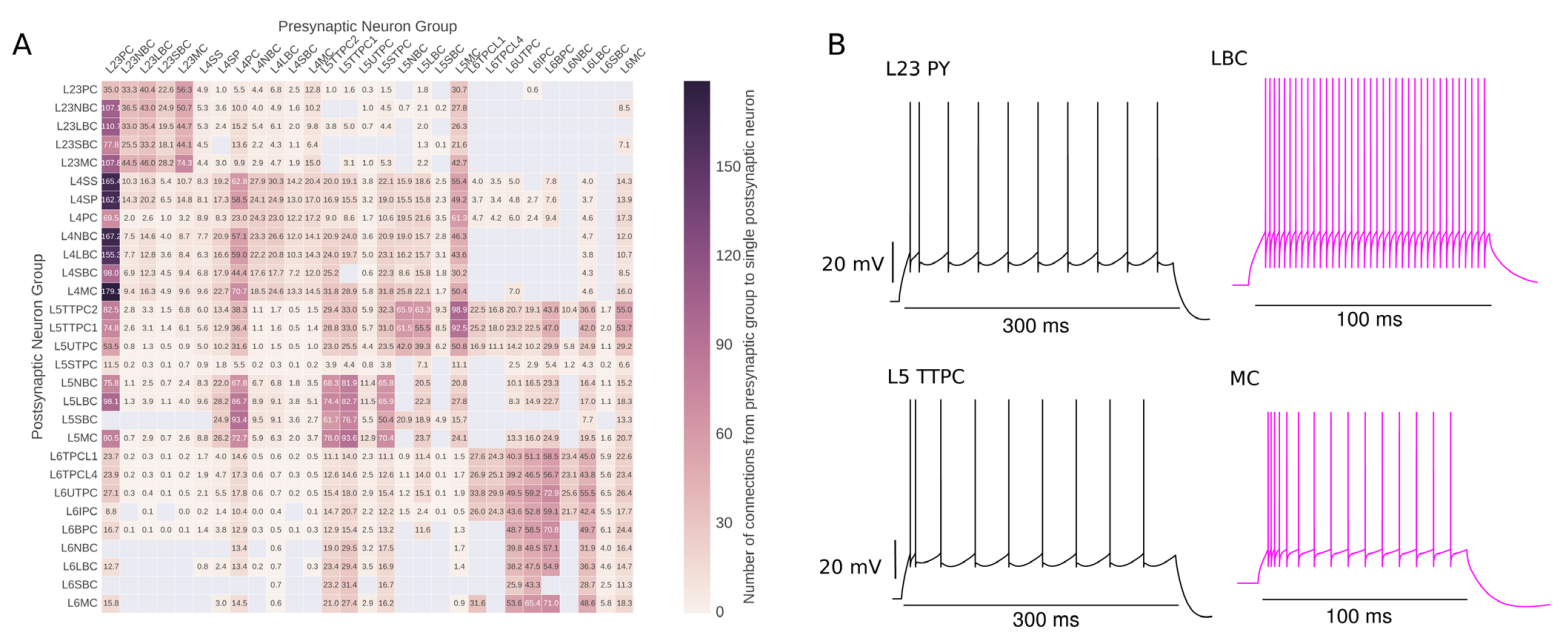
( **A**) The expected number of connections from the population of presynaptic neurons in the presynaptic group to a single neuron in the postsynaptic group. ( **B**) The response of a selection of neuron types to current injection. The amplitudes of injected current were: 500 pA for the L23 PY, 400 for the LBC, 1000 pA for the L5 TTPC, and 400 for the MC. The adaptive exponential integrate and fire model resets at a given threshold Vt, we have extended the trace to 30 mV for illustrative purposes.

The number of connections between neuron groups is shown in Figure 8A, we can see the strong connectivity from layer 2/3 pyramidal cells to all cells in layers 2 to 5. We can also see that other neuron types tend to preferentially synapse onto and receive synapses from neurons in their own layer. Like 2 and 30 we use a 2D Gaussian spatial profile to calculate the probability of connection with increasing distance from the presynaptic neuron in the *X* and *Z* planes. In the *Y* plane, the connection probability is constant. The mean and standard deviation are set using estimates of the axonal arbourisation adapted from 31. Figure 8B shows the response of a selection of neuron types to current injections. We can see regular-spiking pyramidal cells (L23 PY, L5 TTPC), low threshold-firing Martinotti cells (MC), and fast-firing basket cells (LBC).

### The stimulation model

**Figure 9.**
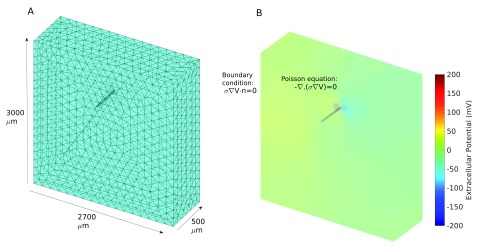
Modelling the field potential generated by a bipolar electrode. ( **A**) The 3D mesh. The initial geometry was built using Blender (essentially a cuboid representing the tissue with the two cuboidal electrodes cut out). The geometry is then meshed using the PDE toolbox. ( **B**) The potential field calculated using the PDE toolbox. The Poisson equation is used as the volume conductor equation, Neumann boundary condition (no current flowing) has been used for the boundary of the tissue/extracellular fluid with the air or recording chamber. The Dirichlet boundary condition has been used for the electrodes. Where *V* = *A*, where *A* is the amplitude of the stimulation in mV and will be positive at one electrode and negative at the other.

We model a bipolar electrode with 10 x 10 *µm* shafts 25 *µm* apart ( Figure 9). We import this model into MATLAB’s PDE Toolbox and use this to construct a finite element model of the electric potential in the slice as a result of a potential difference between the electrodes. Following 32 we model our system as a direct current between two dipoles in a conductive media. We use the Poisson equation for our volume conductor equation, where *σ*, the conductivity, is the only parameter and set at 0.3 S/m. Neumann boundary conditions are used to model the boundary of the tissue/extracellular fluid with the air or recording chamber. This implies that no current will flow across this boundary. The Dirichlet boundary condition is used for the electrode-tissue boundary, where the potential is set to the stimulation amplitude on one electrode and the negation of this on the other.

−∇⋅(σ∇V)=0(24)

σ∇V⋅n=0(25)

We set the amplitude to 750 mV on one electrode and -750 mV on the other. We can then get the current that this corresponds to by calculating the spatial integral of the current density at the boundary of one of the electrodes. In this case we get a current of 54 *µA*.

### Response to stimulation

**Figure 10.**
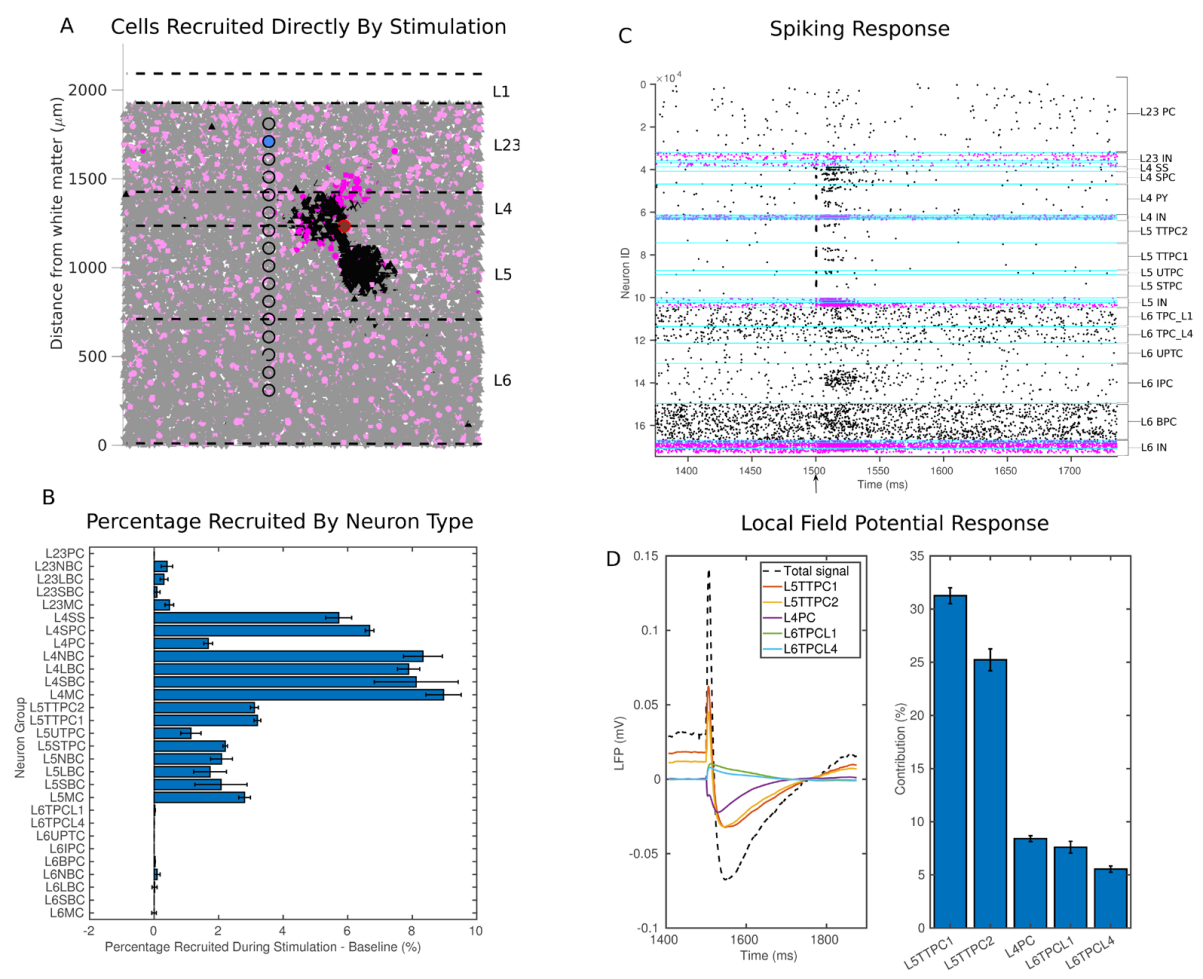
The response to stimulation. ( **A**) Shows the spatial extent of the cells directly recruited by stimulation in a simulation run (black cells excitatory, pink cells inhibitory), the relative positions of the stimulating electrode - the red circle, and recording electrodes - the black circles (the one filled with blue is used for subsequent figures). ( **B**) Shows the extent to which each neuron group is recruited directly by stimulation, showing mean and standard deviation for 5 runs. ( **C**) A spike raster showing the result of a single simulation, the time of stimulation is shown by the arrow on the x axis. ( **D**, left) The LFP recorded at the blue electrode in shown in ( **A**) decomposed into the traces of the 5 neuron groups that contribute most. ( **D**, right) The contribution of each group as a percentage of the total LFP.

**Figure 11.**
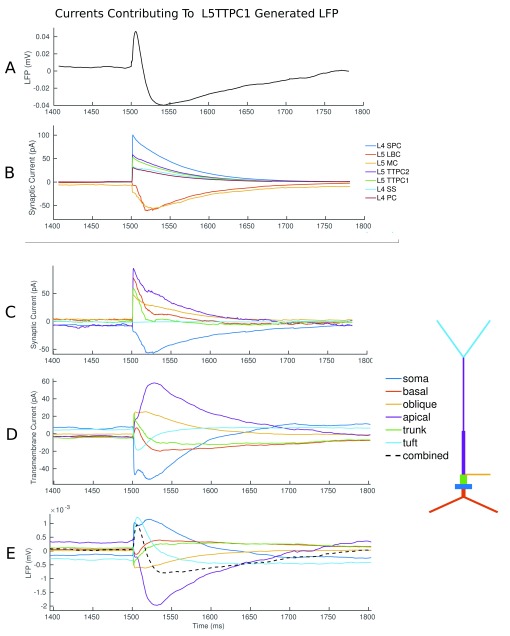
The currents contributing to the LFP generated by the L5TTPC1 neuron group as a result of stimulation. ( **A**) The mean LFP generated by 50 L5TTPC1 neurons near to the recording electrode. ( **B**) The mean synaptic current received by these neurons coloured according to the presynaptic neuron group. ( **C**) The mean synaptic current received by each section of the neuron. Colours correspond to the diagram illustrating the anatomy of the neuron type. ( **D**) The total transmembrane current for each section. ( **E**) The LFP contribution of each section, with the total LFP contributed (dashed line).

Before considering the response to repeat stimulations we first look at the response to a single stimulation. Here we can focus on revealing the neuron populations that generate the LFP response (those neurons whose membrane potential change contributes most significantly to the LFP), and then we can identify those neuron groups whose synapses produce this change. We stimulate using the model outlined above, with a 500 *µs* pulse applied 1500 ms into the simulation. As the generation of the synaptic parameters in our network is stochastic, we generate 5 networks, and apply the stimulation to each. Figure 10A and B illustrate the recruitment of cells during a representative single run, Figure 10C and D show the mean and standard deviation of the 5 runs. From 10 A, we can see that cells may be recruited by stimulation 300 *µm* from the stimulating electrode in the vertical dimension, or 150 *µm* in the horizontal dimension. From Figure 10B we can see that neurons from layers 4 and 5 are well recruited, with between 1 % and 10 % recruited above the baseline. We see that interneuons in layer 4 are particularly receptive - this may be a result of them having a lower threshold for firing as their smaller dendritic structure lends them to be less affected by stimulation in terms of membrane potential change. From Figure 10D we can see that, even when the recording electrode is placed in layer 2/3, the neuron groups contributing most heavily to the LFP are those in layers 4, 5, and even 6, with the thick tufted layer 5 pyramidal cells contributing over 55% of the total signal. They are a large group (14% of the entire model), they receive a large number of synapses from those groups recruited by the stimulation (see connectivity matrix), and they also extend their apical dendrites well into layer 2/3, allowing them to influence the LFP recorded there. Having identified L5TTPCs as a significant source of the LFP, we can then investigate the presynaptic origins of the currents that contribute to the LFP they generate. This will allow us to bridge the gap between what we know of the direct recruitment ( Figure 10A–C) and the local field potential recorded at a particular point - Figure 10D. Figure 11 illustrates the average currents flowing across the 50 L5TTPC1 cells nearest to the recording electrode. The local field potential contributed by these cells is shown in A, with the stimulation artefact removed. Below this we can see the synaptic currents received, coloured according to their presynaptic neuron group. This shows a sharp spike of excitatory current from those L4 and L5 pyramidal and spiny stellate cells (L4SPC, L5TTPC1, L5TTPC2, L4SS, L4PC) directly recruited by stimulation. This is followed by an inhibitory current from the soma-targeting basket cells (LBC) and dendrite-targeting Martinotti cells (MC). When we look to the synaptic current flowing across the various sections of the neuron ( Figure 11C) we see a strong and sharp initial excitatory current at the dendrites. This is followed by an inhibitory current at the soma (mediated by the soma targeting LBCs), and a lower net excitatory current at the dendrites—a result of the initial excitatory barrage decaying combined with indirectly recruited dendrite targeting interneurons (MCs) and indirectly recruited excitatory cells. To arrive at the LFP (shown in Figure 11E) we must calculate the total transmembrane current for each section (shown in Figure 11D), and then apply to this a function of the distance from the section to the recording electrode. More detail on how VERTEX calculates LFPs is available in previously published work ^2^. Now we can see the tuft contributing significantly to the initial inflection in the LFP (as a result of its proximity to the recording electrode, and mediated through axial currents themselves a result of excitatory currents received at apical dendrites). The apical dendrites then make a significant contribution to the secondary deflection as can be seen by following the purple trace in Figure 10D and E. We can see from 10C that the excitatory synaptic current arriving at the apical dendrites is not enough to account for this on its own, and that the axial current produced by the strong polarisation of the soma also makes a large contribution.

### Paired pulse stimulation

**Figure 12.**
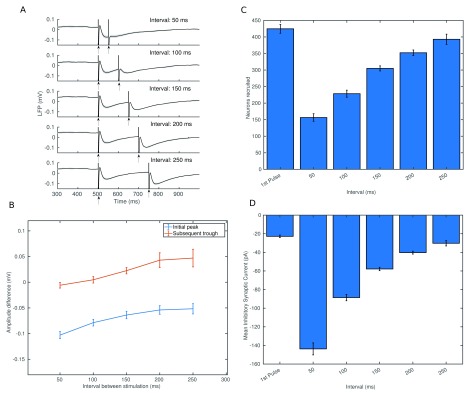
Paired pulse stimulation with an inter-pulse interval of 50 ms, 100 ms, 150 ms, 200 ms, 250 ms. In all cases we show the mean and standard deviation of 5 networks. ( **A**) Shows the local field potential with stimulation artefact included for each inter-pulse interval. Stimulus times are indicated by the arrows. Solid line shows mean, shading indicates standard deviation. ( **B**) Shows the difference in amplitude (second pulse - first pulse) of each component of the response for each interval. ( **C**) Shows the number of L5TTPC cells recruited during stimulation for the initial pulse and for the second pulse for each interval. ( **D**) Shows the mean strength of inhibitory current received by the 50 L5TTPC cells nearest the stimulating electrode at the point of stimulation for the first pulse and then the second pulse for each interval.

**Figure 13.**
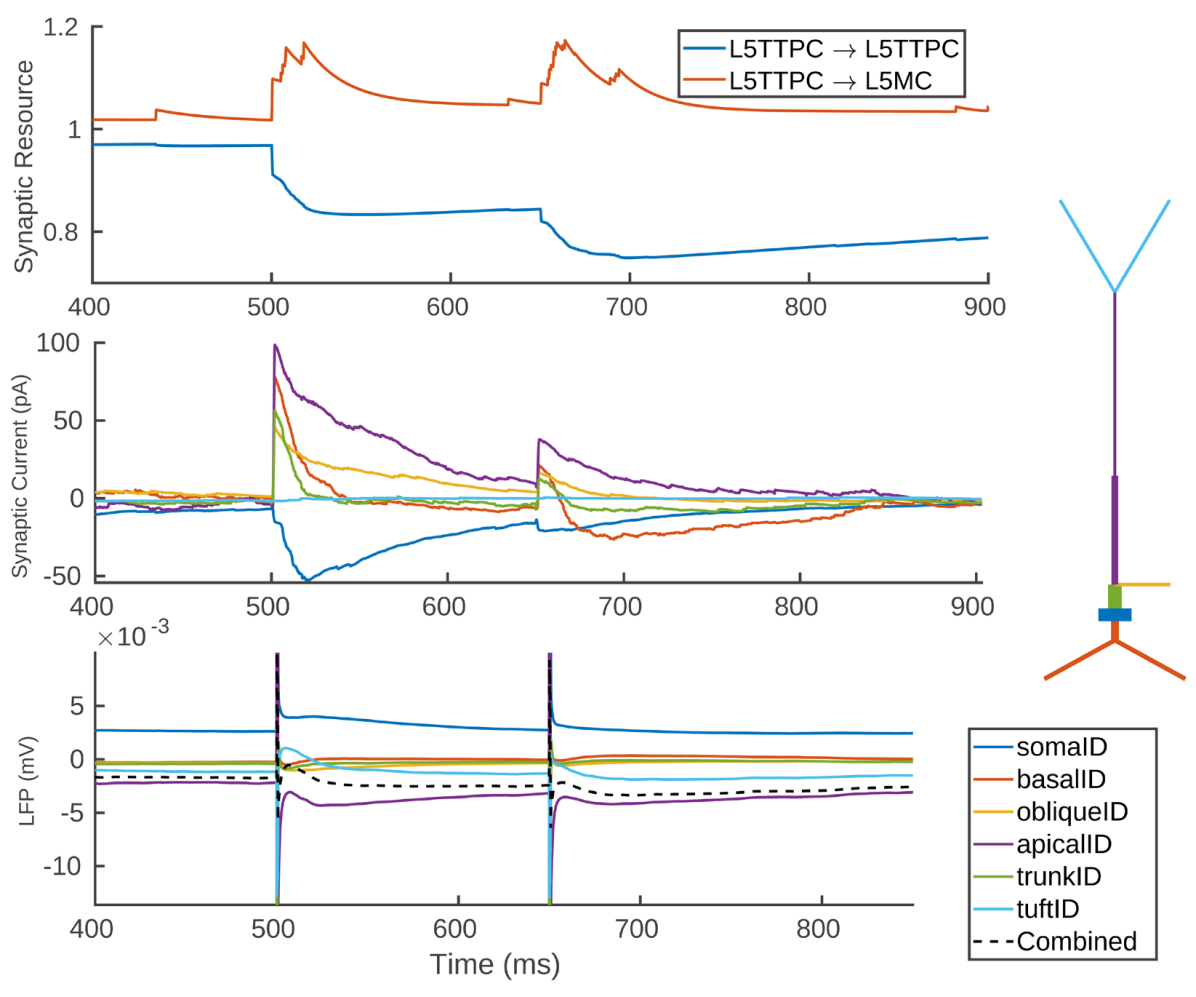
The contribution of short term plasticity to the depression in the response to paired pulse stimulation with an interval of 150 ms. (Top) Shows the synaptic resource available (the resource in the recovered state ( *x*)) that would be applied if there were to be a spike ( *x uU *(1*−u)*) relative to the baseline, for 100 L5TTPC cells nearest the stimulating electrode. The blue line indicates synaptic resources to other L5TTPCs, red to MCs. (Middle) Synaptic current received by each section of the L5TTPC neuron, average for 50 neurons nearest the recording electrode. (Bottom) Average LFP contribution of each section of the 50 L5TTPC neurons nearest the recording electrode.

Having uncovered the main currents contributing to the LFP, we can now consider the contributions to changes in these currents after repeated stimulation. Although short term synaptic plasticity plays a major role in paired pulse depression or facilitation we must also consider factors unrelated to synaptic plasticity. One such important factor is residual inhibition, which increases the threshold required for stimulation in some neurons, reducing recruitment. In Figure 12A and B we can see that for short intervals the response to the second pulse is significantly weakened, but that it recovers as the interval increases. Figure 12C shows us that the number of L5TTPC cells directly recruited by stimulation is more than halved for short intervals but that by 250 ms it has recovered to near 100% of that of the initial pulse. Figure 12D shows us that the residual inhibition follows a similar pattern, with strong residual inhibition at an interval of 50 ms decaying to around baseline after 250 ms. This indicates that residual inhibition plays a significant role for short intervals, but as the number of neurons recruited recovers to around baseline after 200 ms, if we still see significant paired pulse depression on the initial peak and facilitation on the subsequent trough, we can look also to the role of short term plasticity. In Figure 13 we can see the average synaptic resource available on synapses from L5TTPCs to other L5TTPCs (mediating excitatory dendritic currents) and to MCs (mediating inhibitory dendritic currents). We see that the synaptic resource available for excitatory cells is *decreased* for the second pulse, while for the inhibitory cells it is *increased*. This is then reflected in the synaptic currents received on various sections of L5TTPCs near the recording electrode, with the net current arriving at the dendrites much reduced or even negative for some sections. This results in the LFP losing most of its initial peak and its subsequent trough being deeper than before.

### Theta burst stimulation

**Figure 14.**
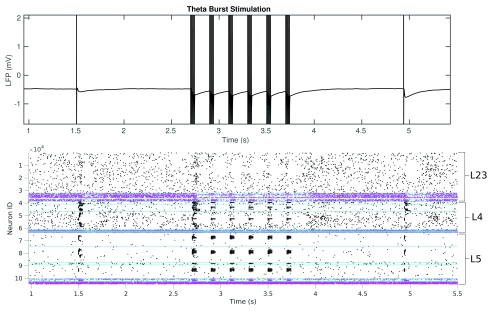
(Top) The effects of TBS on the LFP. (Bottom) The effect of TBS on neuronal activity in terms of neuron spiking. Shows the activity in layers 2/3, 4, and 5 over the course of the protocol. Black dots indicate excitatory neuron spike pink dots indicate inhibitory neuron spike.

**Figure 15.**
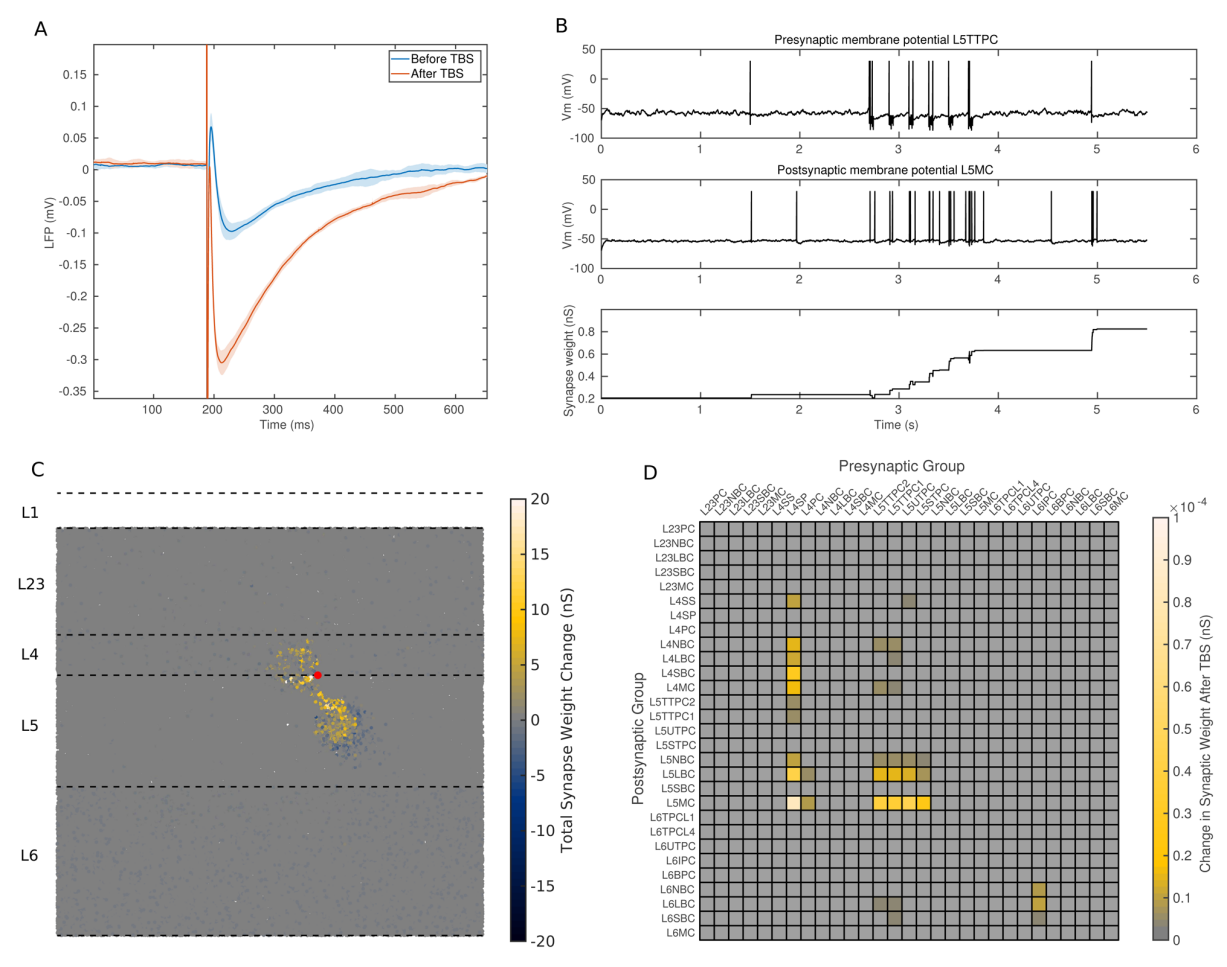
TBS causes a potentiation of the connection between excitatory cells and Martinotti cells. ( **A**) The LFP response recorded in layer 2/3 before TBS (blue) and after TBS (red), showing a potentiation of trough. Lines show the mean, shading shows the standard deviation, from 5 networks. ( **B**) Potentiation of a single L5TTPC to L5MC connection as a result of spike timing dependent plasticity. Shows the increase in synaptic strength (Bottom) brought about by the stimulus recruiting the pyramidal cell (Top) which then causes the Martinotti cell to fire (Middle). ( **C**) The spatial extent of synaptic change brought about by stimulation in a single network. Colour represents the magnitude of synaptic change for each neuron. The red dot shows the location of the stimulating electrode. ( **D**) The change in synaptic weight per group to group connection in a single network. We can see that the change is predominantly an increase in the strength of excitatory to inhibitory connections.

Repetitive stimulation is often used to invoke various forms of long term synaptic plasticity such as LTP (long term potentiation) and LTD (long term depression). The order and timing of input has been shown to be of critical importance when inducing LTP in a particular connection. The connection must be activated within a time window (usually around 20 ms) before the postsynaptic site has been activated. Long term depression (LTD) occurs when the connection is activated after the postsynaptic site and manifests as a decrease in synaptic efficacy. Although it does not capture all processes that lead to LTP or LTD ^33^, it is thought that the spike-timing dependent plasticity rule described above does capture some of the conditions which lead to long term synaptic changes ^34^. The tetanic stimulation used to induce LTP varies; however, theta burst stimulation (TBS) has been shown to induce LTP in hippocampus and neocortex ^35^. Its mechanism of action in the hippocampus has been thoroughly characterised ^36^. In neocortex it has been shown to produce LTP when applied to the connection from layer 4 to layer 2/3; however, this protocol is not utilised as often as others due to the difficulty in interpreting the results ^35^. Here we model TBS induced LTP in neocortex, applying stimulation to layer 4 using the bipolar electrode. We ensure that spike timing dependant plasticity is present on excitatory synapses, using the SynapseModel_g_exp_mt_stdp, which extends the SynapseModel_g_exp, STPModel_mt, and STDPModel. The parameters used are shown in Table 2, we use a rate of 0.05 nS, chosen as a biologically plausible rate of a similar order of magnitude to that used by 10, 37. Values for *τ _pre_* and *τ _post_* reflect estimates taken from 38. We apply an initial pulse to sample the baseline response to stimulation, then apply the TBS, before sampling the response again. The TBS consists of 6 bursts of pulses, each 150 ms apart. Each burst consists of 5 pulses, with a pulse width of 0.5 ms, and a pulse interval of 10 ms. We can see the pattern of stimulation artefacts in Figure 14, the rhythmic nature is reflective of the hippocampal theta rhythm, from where it is derived. From the spike raster we see that the majority of the cells recruited by stimulation, both directly and indirectly are in layers 4 and 5, with the effect on layer 2/3 being a suppression of activity as a result of inhibitory neuron recruitment. As for the paired pulse and single pulse experiments, we generate 5 networks and run the stimulation on each. The averaged responses (before and after TBS) can be seen in Figure 15A. TBS produces a slight depression of the initial peak, but a strong potentiation of the subsequent trough. Our analysis of the synaptic source of the trough tells us that this could be caused by the dendrite targeting inhibitory MCs dominating the excitatory synaptic current. From Figure 15C we can see that cells that are well recruited by stimulation show large synaptic changes (compare this to Figure 10A). When we look at the broad changes in synaptic strength that occur ( Figure 15D) we can see that the strength of connection from the primary cells recruited by stimulation (L4SPC L4PC, L5TTPC) to the MCs is well strengthened. This strengthened connection contributes to greater recruitment of MCs and so to a greater inhibitory current onto the apical dendrites of the large layer 5 pyramidal cells. This larger inhibitory current results in the larger trough seen in the response. The stronger connection also results in faster recruitment of the MCs, shortening the initial peak.

**Table 2.** The STDP parameters used during the theta burst stimulation simulation.

Presynaptic Group	Synapse Type	Rate (nS)	*τ _pre_* (ms)	*τ _post_* (ms)
Superficial Excitatory (L2-4)	g_exp_mt_stdp	0.05	25	75
Deep Excitatory (L5-6)	g_exp_mt_stdp	0.05	25	25
Inhibitory	g_exp_mt	NA	NA	NA

## Discussion

### Comparison with other tools

Several simulation packages offer similar capabilities to VERTEX. The Brian simulator offers an easy to use and adaptable simulation package written in Python ^39^. In particular it has good support for synaptic plasticity, both short term plasticity models and spike timing dependent plasticity models. It is primarily designed for simulating point neurons, and does not have good support for including morphology. This rules out explicit support for any form of electrical stimulation based on the neuron morphology, although electrical stimulation in the form of current injection can be easily incorporated. It also rules out a biophysical model of the LFP based on synaptic currents, as this is also dependent on a morphological neuron.

The Neuron simulator ^40^ is designed to be used for modelling the neuron morphology, channels, and synapses in detail. As such it supports electrical stimulation through its extracellular mechanism, and LFP simulation through the LFPy tool ^41^. It also supports a wide variety of synapse types including short term plasticity and spike timing dependent plasticity. However, the Neuron simulator is not considered easy to use—it is designed for simulating single neurons or small networks in detail and it can become cumbersome for those wishing to simulate large multi-layered networks. VERTEX fulfills the need for an easy to use neural simulation environment focussing on modelling multi-layered structures such as neocortex, LFP generation, synaptic plasticity, and electric field stimulation.

### Comparison with experimental data

We make a prediction of the LFP response measured in layer 2/3 when a stimulus is applied to layer 4 of rat neocortex. We reveal that the main sources of this signal are synaptic currents applied to large layer 5 pyramidal cells, which produce a short peak in LFP coinciding with excitatory synaptic currents, and a subsequent trough coinciding with a drop in excitatory current along with strong inhibitory dendritic currents. This contradicts previous experimental results and established wisdom regarding this paradigm. Previous experimental work tends to assume a purely monosynaptic event with excitatory current contributing to a short peak or trough (of a similar width to our initial peak) in the LFP response ^35^. It is also assumed that synapses onto pyramidal cells in layers 2 and 3 make the greatest contribution, while our model predicts that the apical dendrites of layer 5 pyramidal cells will make the greatest contribution. The significant secondary recruitment of inhibitory neurons in our model results in the large trough that differentiates our results from those of experimentalists ^35^. A possible explanation for this is that our model has been parametrised to more readily recruit the surrounding inhibitory neuron population - a stronger synaptic connection or lower threshold to firing are both possible. The dominance of layer 5 neurons in the generation of the LFP can also partly be explained by this large inhibitory recruitment—inhibitory neurons do not tend to project outside of their layer and pyramidal cells from the layers stimulated will receive the largest currents from them. When we look only at the excitatory component of the LFP, the contribution of the layer 2/3 pyramidal cells is of a similar magnitude to that of the layer 5 pyramidal cells. A further contribution to this discrepancy may be the lack of axonal segments in our model—stimulation in layer 4 is thought to recruit many of the axons projecting into layer 2/3 without recruiting the soma. This would result in a preference for activating excitatory connections onto layer 2/3 pyramidal cells. Discrepancies between our simulated output and that measured experimentally can be fed back into the model building process to produce more accurate connectivity maps. The construction of maps of the neocortical microcircuit is an ongoing field of study ^11, 27– 29, 42^. Constructing accurate maps is seen as a vital step towards facilitating *in silico* research in a variety of areas of neocortex research. From understanding the information processing abilities of healthy neocortex, to understanding its role in various neurological disorders. Discrepancies do not necessarily invalidate the map, as we have mentioned there are other possible sources error. However, simulations built using VERTEX could aid in validation of microcircuit maps by describing the functioning of the network in a specific context with inputs (electrical stimulation) and outputs (LFP) directly comparable with experimental data. This and its ease of use may also make constructing comparison simulations more appealing to experimentalists.

### Using VERTEX to determine the source of paired pulse depression

Paired pulse depression, as commonly observed in neocortex, is a product of at least two processes: the residual inhibition present after the first stimulus suppressing the recruitment during the second ^43^; and the activity dependent plasticity present at the synapses of those cells recruited by stimulation ^20^. These overlapping processes can make interpretation of the results of paired pulse experiments difficult, as in many cases one may want to attribute the response suppression to a single mechanism. One way to overcome this when studying short term plasticity, is to stimulate a single neuron and record intracellularly from another neuron onto which the first synapses. This can reliably tell us the relationship between presynaptic activity and synapse strength, with this type of technique used to generate the data on the NMCP ^11^. However, these experiments are difficult and time consuming to perform, and the variability between individual synapses can be high requiring many repeats. Field stimulation on the other hand, allows one to sample many synapses at once, either on to a single neuron by recording the postsynaptic current intracellularly or onto a population of neurons by recording the LFP response. If one can reliably interpret the results this can make for a more robust technique for those seeking to study the short term plasticity on a specific connection. VERTEX can assist in this by synthesising our knowledge of the underlying circuits to predict which connections are generating the LFP response and what any expected synaptic change would look like. Those looking at the response in a single cell can use VERTEX to predict and then dissect out the presynaptic source of the currents measured.

## Conclusions

We have described the implementation of an extension to the VERTEX simulator ^2^. It now includes the option to use electric field stimulation and synaptic plasticity (both short term and spike timing dependent), making it ideal for simulating full scale models of an *in vitro* cortical slice displaying phenomena such as paired pulse depression or long term potentiation. However, it also opens up the opportunity to model non-invasive brain stimulation in the future. We have described the data structures required to implement this, as well as the workflow involved in building the simulations. We have described an example simulation of rat neocortex, applying electric field stimulation to layer 4 and measuring the response in layer 2/3. We use this to illustrate how the tool can be used to unveil the currents contributing to the field potential response to electrical stimulation. To illustrate the utility of the tool in experiments involving synaptic plasticity and field stimulation, we then have applied two common stimulation paradigms. Applying paired pulse stimulation, we show how the tool can be used to unveil how short term plasticity and residual inhibition affect the response to a second pulse. Applying theta burst stimulation, we show how the tool can be used to estimate the specific connections that will be strengthened during long term potentiation. It is hoped that VERTEX and this model of stimulation in rat neocortex can assist other researchers in investigating the precise synaptic contributions to response field potentials.

## Data availability

The data used to generate the simulations is stored in the ratSo-matosensoryCortex folder on the source code repository: https://github.com/haeste/Vertex_2

The simulation results used to generate the figures in the report can be found at: https://doi.org/10.5281/zenodo.2539398

License: https://creativecommons.org/licenses/by/4.0/legalcodeCC-BY4.0

## Software availability

**Source code available from:** https://github.com/haeste/Vertex_2.

**Archived source code at time of publcation:** https://doi.org/10.5281/zenodo.2543399 ^12^.

**License:** https://github.com/haeste/Vertex_2/blob/master/license.txt.
