# Supplementary material for: The Virtual Electrode Recording Tool for EXtracellular Potentials (VERTEX) Version 2.0: Modelling in vitro electrical stimulation of brain tissue
Source: Wellcome Open Res. 2019 Feb 1;4:20. [Version 1] doi: 10.12688/wellcomeopenres.15058.1 (PMC6439485; doi:10.12688/wellcomeopenres.15058.1)
Supplement: Peer review history

# Peer review: Reviewer response for version 1

**DOI:** 10.21956/wellcomeopenres.16428.r34775

**Published:** 2019-03-27

**Authors:** Gaute T. Einevoll

**Affiliations:** 1 Department of Physics, University of Oslo, Oslo, Norway

The paper of Thornton *et al.* presents a second version of the tool VERTEX (version 2.0). The first version of VERTEX focused on the generation of LFP signals from a patch of brain tissue. In this new version the tool has been extended to include the possibility to model (i) electrical stimulation in a slice and (ii) synaptic plasticity effects (both short-term plasticity and spike-timing dependent plasticity (STDP). As tools like VERTEX will be important to link network models to experimental observations, I think it could be of great value to the community. As the tool also seems technically sound, I support the indexing of this paper.

 There are still some quirks in the presentation could be improved, I think. In particular:

- In Figure 3 the formula describing the electrical potential (not field) around a point source is incomplete (a slash is missing). 
- Page 9, right column: How are dendrites that accidentally end up inside the bipolar electrode treated?
- Page 9, right column: What is the boundary condition for V at the slice boundary?
- Page 9, right column: What is the size of the individual electrode contacts?
- Figure 6 shows stick neurons, while reconstructed neurons are used in the example calculations?
- Page 10, left column: “…that neurons are well-recruited”. Specify what “recruited” means here.
- Figure 9: Maybe describe in the figure caption where the electrode is and where the two (?) contacts are?
- Figure 10A: Here I couldn’t locate any red circles.
- Figure 10D: How is the LFP response in the right panel measured?
- Figure 11A: What does “neurons near to the recording electrode” mean precisely?

To improve the presentation further, I suggest the authors go through the entire manuscript one more time with the eye to increase the precision and clarity of the text.

 It seems to me that the “Comparison with the experimental data” section could be expanded further, for example, by comparing findings with results from other similar modeling projects such as Reimann *et al.*, 2013 ^1^; Markram *et al.*, 2015 ^2^; and Hagen *et al.*, 2016 ^3^. It would also be interesting to see the findings compared with the combined experimental and modeling study on rodent barrel cortex of Einevoll *et al.*, 2007 ^4^.

 Finally, the present reference to LFPy is incomplete (author names are missing). More importantly, a reference to the most recent version of LFPy (LFPy2.0), that is, Hagen *et al.*, 2018 ^5^ should be added.

I have read this submission. I believe that I have an appropriate level of expertise to confirm that it is of an acceptable scientific standard.

## References

[1] ReimannMWAnastassiouCAPerinRHillSLMarkramHKochC: A biophysically detailed model of neocortical local field potentials predicts the critical role of active membrane currents. *Neuron*.2013;79(2) : 10.1016/j.neuron.2013.05.023 375-90 10.1016/j.neuron.2013.05.023 23889937PMC3732581

[2] MarkramHMullerERamaswamySReimannMWAbdellahMSanchezCAAilamakiAAlonso-NanclaresLAntilleNArseverSKahouGABergerTKBilgiliABuncicNChalimourdaAChindemiGCourcolJDDelalondreFDelattreVDruckmannSDumuscRDynesJEilemannSGalEGevaertMEGhobrilJPGidonAGrahamJWGuptaAHaenelVHayEHeinisTHernandoJBHinesMKanariLKellerDKenyonJKhazenGKimYKingJGKisvardayZKumbharPLasserreSLe BéJVMagalhãesBRMerchán-PérezAMeystreJMorriceBRMullerJMuñoz-CéspedesAMuralidharSMuthurasaKNachbaurDNewtonTHNolteMOvcharenkoAPalaciosJPastorLPerinRRanjanRRiachiIRodríguezJRRiquelmeJLRössertCSfyrakisKShiYShillcockJCSilberbergGSilvaRTauheedFTelefontMToledo-RodriguezMTränklerTVan GeitWDíazJVWalkerRWangYZaninettaSMDeFelipeJHillSLSegevISchürmannF: Reconstruction and Simulation of Neocortical Microcircuitry. *Cell*.2015;163(2) : 10.1016/j.cell.2015.09.029 456-92 10.1016/j.cell.2015.09.029 26451489

[3] HagenEDahmenDStavrinouMLLindénHTetzlaffTvan AlbadaSJGrünSDiesmannMEinevollGT: Hybrid Scheme for Modeling Local Field Potentials from Point-Neuron Networks. *Cereb Cortex*.26(12) : 10.1093/cercor/bhw237 4461-4496 10.1093/cercor/bhw237 27797828PMC6193674

[4] EinevollGTPettersenKHDevorAUlbertIHalgrenEDaleAM: Laminar population analysis: estimating firing rates and evoked synaptic activity from multielectrode recordings in rat barrel cortex. *J Neurophysiol*.2007;97(3) : 10.1152/jn.00845.2006 2174-90 10.1152/jn.00845.2006 17182911

[5] HagenENæssSNessTVEinevollGT: Multimodal Modeling of Neural Network Activity: Computing LFP, ECoG, EEG, and MEG Signals With LFPy 2.0. *Front Neuroinform*.2018;12: 10.3389/fninf.2018.00092 92 10.3389/fninf.2018.00092 30618697PMC6305460

# Peer review: Reviewer response for version 1

**DOI:** 10.21956/wellcomeopenres.16428.r34774

**Published:** 2019-03-04

**Authors:** Matthias H. Hennig

**Affiliations:** 1 Institute for Adaptive and Neural Computation, School of Informatics, University of Edinburgh, Edinburgh, UK

This paper describes an extension of a previously published software package for simulating extracellular potentials generated by networks of neurons, which makes it possible to model the effects of electrical stimulation. Neurons are based on integrate and fire models, and morphologies are modelled by the compartmentalised cable equation. Synapses are modelled through conductances, and short term and spike timing dependent plasticity are included. The effects of electrical stimulation are then modelled by computing the field generated by a given electrode configuration in each compartment, and included as a current to the membrane equation. Simulations then show how the simulator can be used to assess protocols such as paired pulse or tetanus stimulation.

 The paper is easy to follow, and gives a good summary of the software. However I would like to make a few suggestions:

1. I suppose this model was specifically built for scale, to enable the simulation of large networks. This, of course, required some simplifications. One issue that would, I think, be relevant to discuss, are the potential effects of stimulation on voltage-gated channels, which cannot be accounted for by the model. Do you have an intuition what difference this may make? I expect it to be more pronounced during high frequency stimulation. 
2. The model assumes a homogeneous medium to compute the effect of stimulation. I think it is, at best, controversial that this is a good assumption (see e.g. Bédard *et al.,* 2004 ^1^). Do you think this is relevant for the stimulation model? Is there perhaps an experiment that could be suggested based on simulations that address this?
    
3. It would be useful to get a better feel for the computational performance of the simulator. Could you include run times of the simulations mentioned under 'Requirements'?
4. The writing is good and clear, but another pass would remove a few rough edges, e.g.:  

- page 5, top left: "object-orientated" -> "object-oriented".
- page 18, left: "from those of experimentalists" -> "experimental results".

I have read this submission. I believe that I have an appropriate level of expertise to confirm that it is of an acceptable scientific standard.

## References

[1] BédardCKrögerHDestexheA: Modeling Extracellular Field Potentials and the Frequency-Filtering Properties of Extracellular Space. *Biophysical Journal*.2004;86(3) : 10.1016/S0006-3495(04)74250-2 1829-1842 10.1016/S0006-3495(04)74250-2 14990509PMC1304017
